# Safety of alternate day fasting and effect on disordered eating behaviors

**DOI:** 10.1186/s12937-015-0029-9

**Published:** 2015-05-06

**Authors:** Kristin K Hoddy, Cynthia M Kroeger, John F Trepanowski, Adrienne R Barnosky, Surabhi Bhutani, Krista A Varady

**Affiliations:** Department of Kinesiology and Nutrition, University of Illinois at Chicago, 1919 West Taylor Street, Room 506 F, Chicago, IL 60612 USA

**Keywords:** Calorie restriction, Alternate day fasting, Safety, Adverse events, Eating disorder behaviors, Obese adults

## Abstract

**Background:**

Alternate day fasting (ADF; ad libitum intake “feed day” alternated with 75% restriction “fast day”), is effective for weight loss, but the safety of the diet has been questioned. Accordingly, this study examined occurrences of adverse events and eating disorder symptoms during ADF.

**Findings:**

Obese subjects (n = 59) participated in an 8-week ADF protocol where food was provided on the fast day. Body weight decreased (P < 0.0001) by 4.2 ± 0.3%. Some subjects reported constipation (17%), water retention (2%), dizziness (<20%), and general weakness (<15%). Bad breath doubled from baseline (14%) to post-treatment (29%), though not significantly. Depression and binge eating decreased (P < 0.01) with ADF. Purgative behavior and fear of fatness remained unchanged. ADF helped subjects increase (P *<* 0.01) restrictive eating and improve (P *<* 0.01) body image perception.

**Conclusions:**

Therefore, ADF produces minimal adverse outcomes, and has either benign or beneficial effects on eating disorder symptoms.

## Introduction

Alternate day fasting (ADF) is an innovative form of dietary restriction capable of decreasing body weight by 4-8% after 8–12 weeks [1-4]. ADF allows dieters to alternate between a “feed-day” involving ad libitum food intake, and a “fast-day” consisting of 75% energy restriction. Although ADF is effective for weight loss, the safety of the diet has been questioned. In particular, concerns have been raised regarding gastrointestinal disturbances, sleep issues, and problems with energy levels (that may be related to lower blood sugar levels). ADF has also been criticized for potentially increasing disordered eating behaviors and negatively impacting body image perception. Accordingly, this study examined the occurrence of adverse events, changes in disordered eating symptoms, and alterations in body image perception after 8 weeks of ADF in obese subjects.

## Materials and methods

### Subject selection

Obese subjects were recruited from the Chicago area by advertisements. Key inclusion criteria were as follows: age 25–65 y, BMI between 30 and 39.9 kg/m [2], weight stable, previously sedentary, non-diabetic, non-smoker, and no history of cardiovascular disease. The experimental protocol was approved by the Office for the Protection of Research Subjects at the University of Illinois, Chicago, and all volunteers gave written informed consent.

### Study design and diet protocol

An 8-week trial was implemented to test the study objectives. The primary outcome measures were body weight, adverse events, disordered eating symptoms, and body image perception. Total energy expenditure was quantified by the Mifflin equation [5] and appropriate activity factors. All subjects consumed 25% of their baseline energy needs on the fast day (24 h), and ate ad libitum on each alternating feed day (24 h). Subjects were provided with meals on each fast day, and ate ad libitum on the feed day. All meals were consumed outside of the research center. Fast day meals were provided as a 3-day rotating menu, based on American Heart Association (AHA) guidelines [6]. Body weight was measured using a balance beam scale (HealthOMeter, Boca Raton, FL), and body composition was assessed by dual energy X-ray absorptiometry (DXA; iDXA, GE Inc.).

### Adverse event, eating disorder, and body image questionnaires

An adverse event questionnaire was administered at baseline (i.e. before the subjects began the intervention) and post-treatment. Eating disorder symptoms were measured using the Multidimensional Assessment of Eating-Disorder Symptoms (MAEDS). The MAEDS is a validated [7] self-report\ inventory that measures six symptom domains related to eating disorders, including: binge eating, restrictive eating, purgative behavior, fear of fatness, avoidance of forbidden foods, and depression. Body image was assessed by the Body Shape Questionnaire (BSQ). The BSQ is a self-report questionnaire that measures excessive concern about one’s body size and shape [8]. Higher scores on the BSQ indicate greater concerns with body size and shape.

### Statistics

Results are presented as mean ± SEM. A paired t-test was used to assess changes from baseline to post-treatment for continuous variables, while a McNemar’s test was used for categorical variables. Differences were considered significant at P < 0.05. All data was analyzed using SPSS software (version 21.0, SPSS Inc, Chicago, IL).

## Results

### Subject dropouts and weight loss

Seventy-four subjects began the study, and 59 subjects (age 46 ± 1 y) completed the 8-week trial. Reasons for dropouts were as follows: inability to comply with the diet (n = 6), scheduling conflicts (n = 2), personal reasons (n = 4), and not specified (n = 3). Body weight decreased (P < 0.0001) from 93.5 ± 1.4 kg to 89.7 ± 1.5 kg (−4.2 ± 0.3%) after 8 weeks of diet. Fat mass was reduced (P < 0.0001) from 41.3 ± 1.0 kg to 39.1 ± 1.0 kg, and visceral fat decreased (P < 0.001) from 1.2 ± 0.1 kg to 1.1 ± 0.1 kg. Lean mass was also reduced (P < 0.001) from 48.7 ± 1.1 kg to 47.3 ± 1.0 kg.

### Adverse events

Constipation was experienced by 17% of subjects with no change throughout the intervention period (Table 1). There were no reports of diarrhea, and a small proportion of subjects (2%) experienced water retention. Some subjects reported bad breath at baseline (14%), and this value doubled (29%) after 8 weeks, though not significantly (P *=* 0.15). Inability to fall asleep was not reported, though a small proportion of subjects (<10%) were unable to stay asleep. Dizziness and general weakness were reported by some subjects (<15%), with no change after 8 weeks of diet.

**Table 1:** **Adverse events reported with 8 weeks of alternate day fasting**

	Baseline	Post-treatment	P-value ^ 1 ^
Gastrointestinal issues			
Constipation (%)	17	17	1.00
Diarrhea (%)	0	0	1.00
Water retention (%)	2	2	1.00
Bad breath (%)	14	29	0.15
Sleep disturbances			
Inability to fall asleep (%)	0	0	1.00
Inability to stay asleep (%)	7	2	0.50
Other adverse effects			
Dizziness (%)	10	17	0.38
General weakness (%)	14	10	0.69

Values reported as mean % occurrences at each time point (n = 59).

^1^P-value between baseline and post-treatment: McNemar test.

### Eating disorder symptoms and body image perception

Depression and binge eating decreased (P < 0.01) after 8 weeks of ADF (Table 2). Purgative behavior was low at baseline and remained unchanged. Fear of fatness was moderate, and did not change from baseline to post-treatment. The intervention helped subjects increase (P *<* 0.01) restrictive eating, but had no impact on avoidance of forbidden foods. Body image perception improved (P *<* 0.01) from baseline to post-treatment.

**Table 2:** **Changes in eating disorder symptoms and body image perception after 8 weeks**

	Baseline	Post-treatment	P-value ^ 1 ^
Eating disorder symptoms			
Depression	27 ± 1	25 ± 1	0.01
Binge eating	33 ± 1	30 ± 1	<0.01
Purgative behavior	9 ± 1	9 ± 1	0.38
Fear of fatness	43 ± 2	43 ± 1	0.71
Restrictive eating	26 ± 1	31 ± 1	<0.01
Avoidance of forbidden foods	37 ± 2	39 ± 2	0.14
Body image			
Concerns about body size/shape	53 ± 2	48 ± 2	<0.01

Values reported as mean ± SEM (n = 59).

^1^P-value between baseline and post-treatment: Paired t-test.

## Discussion

Subjects undergoing ADF experienced mild gastrointestinal issues, occasional problems with staying asleep, and minor dizziness/weakness. The rate of adverse events reported with ADF appears to be similar to that of daily calorie restriction. For instance, Cuevas et al. [9] and Lin et al. [10] observed minimal adverse outcomes with 30-50% restriction after 4–12 weeks (<15% of participants reported constipation, diarrhea, or dizziness). In the present study, the most commonly reported adverse event was bad breath. However, this side effect may be lessened by consuming more water throughout the day and chewing sugar-free gum between meals.

As for eating disorder symptoms, ADF does not increase the rate depression, binge eating, purgative behaviour, fear of fatness, or avoidance of forbidden foods, and may have small beneficial effects on body image perception. Interestingly, restrictive eating was moderately increased with ADF, suggesting that the diet may help control unrestrained eating behaviors [7]. These findings for ADF are comparable to those of calorie restriction. In a recent study by Williamson et al. [11], it was demonstrated that 25% daily restriction did not increase eating disorder symptoms and had no other harmful psychological effects. Taken together, it is possible that neither ADF nor calorie restriction increase eating disorder symptoms in obese adults, but these findings require confirmation in a longer-term randomized control trial comparing the two diets.

This study has several limitations. Firstly, the adverse event questionnaire was not very comprehensive, and was developed based on adverse events reported by subjects in previous ADF trials conducted by our lab. Assessing the impact of ADF on cold intolerance, hair loss, headaches, muscle cramps, and difficultly concentrating, would provide a more comprehensive portrayal of the diet’s safety profile. Second, the intensity of these adverse events (mild, moderate or severe) were not examined and recorded. Third, much of the adverse event data reported in this study was qualitative, and therefore may underestimate all potential adverse events associated with ADF. However, it should be noted that there is mounting quantitative data from previous studies [1-4] indicating that ADF may protect against the development of cardiovascular and metabolic diseases. Thus, the present data should be evaluated in the context of these previous reports [1-4]. Fourth, this study is limited in that it was short term (8 weeks), had a small sample size (n = 59), and did not directly compare the effects of ADF versus calorie restriction on these endpoints.

In summary, ADF appears to be a safe and effective way to lose weight, and produces little or no gastrointestinal, sleep, or energy level disturbances. We also show here that this diet does not increase eating disorder symptoms, and has benign or beneficial effects on body image perception. These preliminary findings are an encouraging first step in this field, but obviously warrant confirmation in a larger-scale, longer-term clinical trial.
